# Housing Insecurity and Older Adults’ Health and Well-Being in a Gentrifying City: Results from the EPIPorto Cohort Study

**DOI:** 10.1007/s11524-024-00921-4

**Published:** 2024-09-26

**Authors:** Cláudia Jardim Santos, Ana Henriques, Carla Moreira, Ana Isabel Ribeiro

**Affiliations:** 1 EPIUnit-Instituto de Saúde Pública, Universidade Do Porto, Rua das Taipas, 135, 4050-600 Porto, Portugal; 2 Laboratório Para a Investigação Integrativa E Translacional Em Saúde Populacional (ITR), 4050-600 Porto, Portugal; 3 Departamento de Ciências da Saúde Pública E Forenses, e Educação Médica, Faculdade de Medicina, Universidade Do Porto, 4200-450 Porto, Portugal; 4 Centro de Matemática, Escola de Ciências, Universidade Do Minho, 4710-057 Braga, Portugal

**Keywords:** Urban health, Age-friendly cities, Health equity, Gentrification, Social determinants

## Abstract

Using 2022 data from 600 adults (≥ 60 years) in Porto, Portugal, we explored the association between housing insecurity and various health outcomes. We examined housing conditions, affordability, and stability in relation to loneliness, quality of life, cognitive function, perception of healthy ageing, and sleep using regression models. Older adults without house heating (*β* = 2.293; 95%CI = 0.753, 3.833), with leaks/dampness/rot (*β* = 3.741; 1.818, 5.664), insufficient daylight (*β* = 2.787; 0.095, 5.479), living in neighborhoods with noise (*β* = 1.793, 0.280 to 3.305), pollution/grime (*β* = 2.580; 0.746, 4.414), and violence/crime/vandalism (*β* = 3.940; 1.723, 6.157), who faced housing cost overburden (*β* = 2.001; 0.426, 3.577), eviction (*β* = 12.651; 0.852, 24.450), and moved frequently (*β* = 4.129; 1.542, 6.716) exhibited higher levels of loneliness. Similarly, lack of house heating (*β* = − 1.942; − 3.438, − 0.445), leaks/dampness/rot (*β* = − 4.157; − 5.999, − 2.316), insufficient daylight (*β* = − 3.124; − 5.714, − 0.534), noise (*β* = − 2.143; − 3.600, − 0.686), pollution/grime (*β *= − 2.093; − 3.860, − 0.325), violence/crime/vandalism (*β *= − 2.819; − 4.948, − 0.691), and those with housing cost overburden (*β *= − 2.435; − 3.930, − 0.940) reported lower quality of life. Those with no toilet (*β* =  − 1.891; − 3.760, − 0.021) or shower (*β* =  − 1.891; − 3.760, − 0.021) and who faced forced displacement (*β* =  − 2.179; − 3.516, − 0.842) presented lower cognitive function. Furthermore, those living in neighborhoods with pollution/grime (OR = 0.494; 0.322, 0.756) and violence/crime/vandalism (OR = 0.477; 0.284, 0.801), those in social housing (OR = 0.728; 0.575, 0.922), and those who moved frequently (OR = 0.475; 0.257, 0.879) reported lower levels of perceived healthy ageing. Insufficient sleep was more common among residents in social housing (OR = 2.155; 1.102, 4.213), while poor sleep quality was least likely both among those living in social housing (OR = 0.445; 0.220, 0.900) and affordable housing (OR = 0.381; 0.162, 0.896). Good quality, stable, and affordable housing seems crucial for healthy ageing.

**Supplementary Information:**

The online version contains supplementary material available at 10.1007/s11524-024-00921-4.

## Background

Our world’s agendas should prioritize the health and well-being of older adults as their numbers are continuously increasing. Projections indicate that, in 2050, one in six people will be over the age of 65 [1]. Aligned with the United Nations Decade of Healthy Ageing (2021–2030), now is the time to achieve a better life for older adults.

Most older adults live in urban spaces. Globally, over 500 million people aged 65 or over reside in cities, accounting for 58% of all older people [2]. In Europe, 90.4 million people fall into the 65 and older category, with 38% living in urban regions [3], and older people aged 60 + are projected to represent around 35% of the urban population [4]. In Portugal, individuals aged 65 and above exceed 2.5 million, with 21% residing in urban areas [5]. The ageing of urban populations poses challenges in creating residential environments that support the well-being of older adults, who often face a heightened vulnerability to the residential environment due to long-term residence and reduced mobility and financial resources [6].

Housing has long been a focal point in public health, given the adverse health effects of inadequate housing or poor-quality living conditions. Interest in housing and health has resurged in the last few years due to increasing housing costs and inadequate policy responses [7]. According to the 2022 European Union statistics on income and living conditions (EU-SILC), 16.8% of the European Union (EU) population lived in overcrowded households; the share of the EU population unable to adequately heat their homes increased from 6.9% (2021) to 9.3%, and 8.7% spent 40% or more of their household disposable income on housing [8]. From 2010 to 2022, housing prices increased at unprecedented rates in the EU—the average rents increased by 19% and house prices by 47% [9]. Notably, these increases have not affected all countries equally, with Portugal currently facing a severe housing crisis.

Porto (Northern Portugal), after the 2008–2009 economic crisis, faced a severe housing crisis exacerbated after 2013, characterized by skyrocketing housing prices, inadequate public investment, and widespread displacement due to gentrification [10]. Gentrification involves a set of processes of sociospatial change driven by investments in the built environment targeted for affluent people [11], leading to a revitalization of the built environment, displacement of former residents — particularly those with less economic resources — and the arrival of wealthier individuals. Qualitative evidence from Porto suggests mixed health implications: some residents noted positive effects, while others reported poor mental health, increased mortality risk, loneliness, and social isolation due to displacement and housing shortages [11]. Despite improvements in housing conditions, Porto still has unfavorable housing types—such as substandard housing (locally called “ilhas”) and social housing—which are associated to adverse health outcomes, like premature death and unhealthier behaviors [12]. Moreover, national data suggest that forms of housing deprivation, such as poor hydrothermal comfort, building pathologies (e.g., mold, dampness), and buildings needing repair are still prevalent [13], making housing insecurity a critical public health concern again.

Housing insecurity is related to a combination of housing features, which may compromise people’s health if unmet. While there is no consensual definition, this concept has been operationalised in the literature in multiple ways. Housing insecurity usually includes dimensions linked to housing affordability (burden of high housing costs, living with family or friends to share housing costs), housing decency and safety (overcrowding, living in substandard poor quality housing, living in unsafe neighborhoods and lack of access to transportation, jobs, quality schools, and other critical amenities), and stable housing occupancy (evictions, forced moves, moving frequently) [14].

Housing insecurity can contribute to physical and mental health issues and was found to be prevalent (14%) in midlife and ageing populations in the United States of America (USA) [15]. Older renters, particularly, usually rely on fixed incomes that cannot stretch for escalating housing expenses. Balancing housing costs with essential expenses like groceries, appliances, and medication [16] can compromise their quality of life (QoL). In the USA, older adults who experienced housing insecurity experienced lower self-rated physical health and increased occurrence of chronic conditions [17] which can lead to a negative self-assessment of their aging process. In the same country, those facing mortgage difficulties or foreclosure also show lower cognitive scores [18]. Although there is currently no literature specifically examining the impact of housing insecurity on sleep and loneliness in older adults, it has been found that housing payment problems are associated with disrupted sleep and poorer mental health [19], the latter of which may contribute to feelings of loneliness. However, often, the research that focuses on housing and health narrows on homelessness or single aspects of housing insecurity, neglecting broader impacts on general older adult populations [20]. Moreover, in Porto, no studies have comprehensively explored the health impacts of various dimensions of housing insecurity.

Against this background, the present study aimed to investigate the association between housing insecurity and indicators of older adults’ health and well-being living in Porto (Portugal).

## Methods

### Participants

This study uses data from EPIPorto cohort study (https://ispup.up.pt/en/coorte/epiporto-2/), a community-based prospective cohort of non-institutionalized Portuguese adults in Porto. Participants were randomly selected through digit-dialing landline telephone numbers, gathering 2485 people who underwent five follow-up evaluations. The baseline assessment was conducted between 1999 and 2003, with the latest assessment concluding in 2022, involving 836 participants (see Figure S1 in supplementary material).

In this cross-sectional study, we selected people 60 years or older, as it is the United Nations' definition of old age, from the ones who participated in the fifth follow-up (*n* = 600). Characteristics of these participants were compared with those who declined to participate (*n* = 178) and those who were part of the cohort but did not undergo assessment (*n* = 1240), as shown in supplementary table S1. The distribution of sexes was fairly consistent, but non-participants were older (mean age 79 for refusals and 85 for non-participants) compared to participants (mean age 73). Individuals who declined to participate also tended to have lower levels of education (participants: 41.8%, refusals: 62.4%, non-participants: 66.7%). Participants completed a structured questionnaire administered by a trained researcher covering sociodemographic data, sleep, housing characteristics and insecurity, loneliness, quality of life (QoL), and cognitive evaluation. Only variables collected in this evaluation were used.

### Housing Insecurity

To assess housing insecurity, we used the fifth follow-up set of questions related to participants’ housing conditions (availability of house equipment and amenities, presence of problems in the house and neighborhood, overcrowding, and the type of housing, such as substandard housing), affordability (such as high housing costs and forced displacement), and stability (frequent moves and tenure status). These groupings were created for simplicity, but we acknowledge their interdependence. For example, tenure status and forced displacement can be related to both housing affordability and stability.

Regarding housing conditions, we inquired about the availability of certain house equipment and amenities, specifically the presence of a private garden, heating, balcony, piped water, toilet, shower, and washing machine. Moreover, we posed straightforward yes-or-no self-perception questions to the participants about the presence of various problems in the house and neighborhood of residence, including leaking roof, damp walls/floors/foundation, or rot on the windows frames or floor (from now on named “leaks/dampness/rot”); insufficient daylight on a sunny day (from now on named “insufficient daylight”), noise felt in the accommodation from neighbors or the street (from now on named “noise”); pollution, grime, or environmental problems from traffic or industry (from now on named “pollution/grime”); and crime, violence, or vandalism in their local area (from now on named “crime/violence/vandalism”). These questions were selected and translated from the European statistics on income and living conditions (EU-SILC) questionnaire (Module on Environment of the Dwelling). In addition, we evaluated if the participants lived in an overcrowded household by analyzing the number of rooms (bedrooms and living room) and the family composition, considering the occupants’ ages, sexes, and relationships of the occupants following the Eurostat definition. Eurostat defines that a person lives in an overcrowded household when the housing arrangement does not comply with allocating one room per couple, assigning one room per individual aged 18 or older, providing one room for pairs of same-gender single individuals between 12 and 17 years old or individual rooms for those not of the same gender, and assigning one room for each pair of children under 12 years old. Finally, we assessed which type of housing they lived in, in the fifth assessment, (conventional, affordable, social, or substandard housing (“ilhas”) as in a previous study from the EPIPorto cohort [12].

To capture housing affordability, we also inquired about the range of housing costs, namely which percentage of the income was spent on housing (60% or more, 40–59%, 20 to 39%, and less than 20% of the disposable income). This variable was dichotomized into housing costs equal to or higher than 40% (overburden) or less than that, as defined by the Eurostat. Moreover, we included dichotomous questions about forced displacement: “In the past 10 years, have you ever been forced to move or leave your home for any of the following reasons: inability to pay rent/loan (yes/no); the landlord has increased the rent of the house to a very high value (yes/no); I got evicted (yes/no); the lease was not renewed (yes/no).” The research team formulated this question and pertained to the last 10 years, which marked the period during which gentrification and touristification became prominent in Porto.

To assess housing stability, we counted how many times participants moved since entering the cohort, as frequent moves reflect instability. We dichotomized the variable into two categories: ‘‘not moved at all or moved once’ and ‘‘have moved two or more times.” We also included tenure status (owner, renter, lives in family housing, or other) because it may influence housing stability and insecurity.

### Health Outcomes

We assessed the impact of housing insecurity on several health and well-being outcomes: loneliness, quality of life (QoL), cognitive function, perception of healthy ageing, and sleeping habits. These outcomes were selected based on their plausible connection to housing insecurity, as presented in the “Background” Sect. [15–19].

Loneliness was measured using 16 items from the Portuguese version of the University of California, Los Angeles (UCLA) loneliness scale for older adults. This scale uses a four-point rating system (1 = Never; 2 = Rarely; 3 = Sometimes; 4 = Frequently), where the minimum and maximum scores are 16 and 64, and higher scores denote a greater feeling of disconnection from others.

QoL was measured using the 24 items of the Portuguese version of the World Health Organization instrument to assess the quality of life in older adults (WHOQOL-OLD). The self-response items are evaluated using a five-point Likert scale (1 = Extremely; 2 = Very; 3 = Moderately; 4 = Slightly; 5 = Not at all). Higher scores denote greater levels of QoL.

Cognitive function was measured using the mini-mental state examination (MMSE). The MMSE is a cognitive screening test that uses 30 items to assess various areas of cognitive function. The maximum score is 30 points (one point per item); higher scores mean better cognitive function.

We also asked the participants if they agreed or disagreed with the following question: “I’m ageing successfully.” To measure the level of agreement with the statement, they could choose between four options (1 = Strongly agree; 2 = Somewhat agree; 3 = Somewhat disagree; 4 = Strongly disagree).

For sleeping habits, we used the self-reported sleeping hours to categorize our participants as “sleeping less than 7 h” or “sleeping 7 or more hours,” based on the National Sleep Foundation’s sleep time duration recommendations for older adults. To categorize our participants’ sleep quality into “bad sleeping quality” or “good sleeping quality,” we did a latent class analysis (LCA) based on their pattern of answers to the following sleeping habits questions asked in the fifth follow-up: “How many hours, on average, do you sleep in a typical night’s sleep?” “Do you often feel tired, fatigued, or sleepy during the day?” “How often, in the last month, have you had difficulty falling asleep?” “How often, in the last month, have you woken up in your sleep and had difficulty falling back asleep?” and “How often, in the last month, did you wake up before the desired time and could not fall back asleep?” We used LCA to categorize participants into distinct sleep quality groups based on their overall sleep patterns. Groups are characterized in supplementary material Figure S2.

### Covariates

From the fifth follow-up, the self-reported information about participants’ sex (male/female), birth date (used to calculate age), marital status (partnered/non-partnered), education attainment (lower than primary—if they had less than 4 complete years of education; primary—if they had 4 or 6 years of complete education; secondary—if they had 9 or 12 years of complete education; tertiary—if they had a degree after 12 years of education), and current or last professional activity (manual/non-manual—classified into 10 groups according to the Portuguese Classification of Occupations 2010, which aligns with the International Standard Classification of Occupations (ISCO-08) and was further simplified into non-manual (for groups 0–5) or manual (for groups 6–9).

### Statistical Analysis

Categorical variables were presented as absolute frequencies and percentages and continuous variables as the mean and standard deviation. Latent class analysis (LCA) was used to identify subgroups (latent groups) within the sample based on their responses to categorical sleep-related variables. LCA allows the detection of individuals who share common characteristics based on the patterns of responses and, therefore, can be grouped. Crude and adjusted linear regression models were computed to estimate the associations between housing insecurity–related variables and continuous health variables (loneliness, QoL, and MMSE), reported as coefficient (*β*) with 95% confidence intervals (95% CI). The associations for sleeping hours, sleep quality, and housing insecurity were calculated using crude and adjusted logistic regression models. Additionally, we employed ordinal regression to analyze the association between the perception of healthy ageing and housing insecurity, expressed as odds ratio (OR) and corresponding 95% CI. Analyses were performed using SPSS version 29.0 and R software version 4.2.2, using the poLCA library.

## Results

### Descriptive Statistics

As depicted in Table 1, participants had a mean age of 73.1 years, most of whom were female (65.0%), had a partner (64.0%), secondary education (32.2%), and a non-manual professional activity (71.8%) (Table 1).

**Table 1:** Participant characteristics regarding general sociodemographic characteristics and housing insecurity features (EPIPorto Cohort, Porto, Portugal, 2022)

Variable	Counts (%) or mean (range)
*General sociodemographic characteristics*	
Mean age (range)	73.14 (60–99)
Sex	
Female	390 (65.0%)
Male	210 (35.0%)
Marital status	
Partnered	384 (64.0%)
Non-partnered	216 (36.0%)
Education	
Lower than primary	23 (3.8%)
Primary	192 (32.0%)
Secondary	193 (32.2%)
Tertiary	192 (32.0%)
Current or last professional activity	
Manual	138 (23.0%)
Non-manual	431 (71.8%)
Missing	31 (5.2%)
*Housing conditions (equipment and amenities)*	
Private garden	
Yes	114 (19.0%)
No	483 (80.5%)
Missing	3 (0.5%)
Heating	
Yes	283 (47.2%)
No	314 (52.3%)
Missing	3 (0.5%)
Balcony	
Yes	500 (83.3%)
No	97 (16.2%)
Missing	3 (0.5%)
Piped water	
Yes	595 (99.2%)
No	2 (0.3%)
Missing	3 (0.5%)
Toilet	
Yes	593 (98.8%)
No	4 (0.7%)
Missing	3 (0.5%)
Shower	
Yes	593 (98.8%)
No	4 (0.7%)
Missing	3 (0.5%)
Washing machine	
Yes	588 (98.0%)
No	9 (1.5%)
Missing	3 (0.5%)
*Housing conditions (problems in the home and neighborhood of residence)*	
Leaks/dampness/rot	
Yes	103 (17.2%)
No	484 (80.7%)
Missing	13 (2.2%)
Insufficient daylight	
Yes	48 (8.0%)
No	539 (89.8%)
Missing	13 (2.2%)
Noise	
Yes	197 (32.8%)
No	390 (65.0%)
Missing	13 (2.2%)
Pollution	
Yes	105 (17.5%)
No	482 (80.3%)
Missing	13 (2.2%)
Violence	
Yes	70 (11.7%)
No	515 (85.8%)
Missing	15 (2.5%)
Overcrowding	
Yes	21 (3.5%)
No	576 (96%)
Missing	3 (0.5%)
House typology	
Social housing	56 (9.3%)
Affordable housing	26 (4.3%)
Substandard housing (“ilhas”)	14 (2.3%)
Conventional housing	504 (84.0%)
*Housing affordability*	
Housing costs (percentage of the income spent on housing)	
Less than 40%	364 (60.7%)
40% or more per cent	170 (28.3%)
Missing	66 (11.0%)
Forced to move due to inability to pay rent/loan	
Yes	2 (0.3%)
No	581 (96.8%)
Missing	17 (2.8%)
Forced to move because the landlord has increased the rent of the house to a very high value	
Yes	8 (1.3%)
No	575 (95.8%)
Missing	17 (2.8%)
I got evicted	
Yes	2 (0.3%)
No	581 (96.8%)
Missing	17 (2.8%)
The lease was not renewed	
Yes	4 (0.7%)
No	579 (96.5%)
Missing	17 (2.8%)
*Housing instability*	
Residential mobility	
Have moved two or more times	48 (8.0%)
Not moved more than once	552 (92.0%)
House ownership	
Owner	428 (71.3%)
Renter	145 (24.2%)
Lives in family housing	17 (2.8%)
Other	5 (0.8%)
Missing	5 (0.8%)

Regarding their housing conditions and the existence of housing equipment and amenities, most had a balcony (83.3%), piped water (99.2%), toilet (98.8%), shower (98.8%), and washing machine (98.0%). Still, most houses did not have a private garden (80.5%) or heating (52.3%). A substantial number reported having leaks/dampness/rot in their house (17.2%), insufficient daylight (8.0%), noise (32.8%), and pollution/grime (17.5%), or crime/violence/vandalism (11.7%) in the house surroundings. About 3.5% of these older adults reside in overcrowded conditions. The majority lived in a conventional housing type (84.0%).

Regarding housing affordability, a substantial 28.3% allocate 40% or more of their income to housing expenses. Only a small number of participants reported being forced to move or leave their home due to the inability to pay rent/loan (0.3%), having their rent increased to a high value by their landlord (1.3%), being evicted (0.3%), and having their lease not renewed (0.7%).

Regarding housing instability, the vast majority of participants had never moved house or had experienced relocation only once (92.0%), partly because most were homeowners and lived in their own homes (71.3%).

Regarding health outcomes, as shown in Table 2, the mean score for loneliness was 24.86 (± 8.55), and for QoL was 73.36 (± 8.33). The average score for the MMSE was 27.62 (± 2.33). Almost half of the participants (49.0%) answered “Strongly agree” to the statement, “I’m aging successfully”, regarding their perception of healthy ageing. More than half of the participants slept at least 7 h (55.2%); however, most people had a bad sleep quality (65.0%).

**Table 2:** Participants’ characterization regarding health and well-being (EPIPorto Cohort, Porto, Portugal, 2022)

Variable	Mean (standard deviation) or frequencies (percentages)
Loneliness	24.86 (8.55)
Missing	53 (8.8%)
Quality of Life	73.36 (8.33)
Missing	55 (9.2%)
Mini-mental state examination (MMSE)	27.62 (2.33)
Missing	86 (14.3%)
Perception of healthy ageing	
Strongly disagree	12 (2.0%)
Somewhat disagree	34 (5.7%)
Somewhat agree	217 (36.2%)
Strongly agree	294 (49.0%)
Missing	43 (7.2%)
Sleeping hours	
Less than 7 h of sleep	244 (40.7%)
Seven or more hours of sleep	331 (55.2%)
Missing	25 (4.2%)
Sleep quality	
Good sleep quality	185 (30.8%)
Bad sleep quality	390 (65.0%)
Missing	25 (4.2%)

### Associations with Housing Insecurity

As depicted in Table 3, according to the adjusted models, older adults experiencing various forms of housing insecurity exhibited higher levels of loneliness and lower quality of life. Specifically, for those without housing heating (*β* = 2.293, 95% CI = 0.753 to 3.833), with leaks/dampness/rot (*β* = 3.741, 95% CI = 1.818 to 5.664), insufficient daylight (*β* = 2.787, 95% CI = 0.095 to 5.479), neighborhood noise (*β* = 1.793, 95% CI = 0.280 to 3.305), pollution/grime (*β* = 2.580, 95% CI = 0.746 to 4.414) and violence/crime/vandalism (*β* = 3.940, 95% CI = 1.723 to 6.157), housing cost overburden (*β* = 2.001, 95% CI = 0.426 to 3.577), who experienced eviction (*β* = 12.651, 95% CI = 0.852 to 24.450), and moved frequently (*β* = 4.129, 95% CI = 1.542 to 6.716) exhibited higher levels of loneliness. In crude models, living in overcrowded houses was associated with higher loneliness [*β* = 4.921 (1.131 to 8.710)], but this was not significant after adjustment.

**Table 3:** Associations between housing insecurity variables and loneliness, quality of life, and mini-mental state examination: EPIPorto Cohort, Porto, Portugal, 2022

Exposures	Health outcomes					
	Loneliness		Quality of life		Mini-mental state examination	
	Crude *β* (95% CI)	Adjusted *β* (95% CI)	Crude *β* (95% CI)	Adjusted *β* (95% CI)	Crude *β* (95% CI)	Adjusted *β* (95% CI)
Without private garden (ref. no.)	1.297 (− 0.536 to 3.131)	1.441 (− 0.396 to 3.278)	− 0.543 (− 2.326 to 1.241)	− 0.521 (− 2.289 to 1.248)	** − 0.549 (− 1.062 to − 0.036)**	− 0.356 (− 0.791 to 0.079)
Without heating (ref. no.)	**2.661 (1.247 to 4.074)**	**2.293 (0.753 to 3.833)**	** − 2.931 (− 4.307 to − 1.555)**	** − 1.942 (− 3.438 to − 0.445)**	** − 1.081 (− 1.474 to − 0.688)**	− 0.261 (− 0.631 to 0.109)
Without balcony (ref. no.)	0.700 (− 1.255 to 2.656)	1.313 (− 0.684 to 3.310)	0.314 (− 1.611 to 2.239)	− 0.080 (− 2.020 to 1.860)	** − 0.595 (− 1.138 to − 0.052)**	− 0.071 (− 0.540 to 0.399)
Without piped water (ref. no.)	− 0.862 (− 12.715 to 10.991)	− 3.732 (− 15.297 to 7.832)	− 0.447 (− 11.999 to 11. 104)	0.414 (− 10.712 to 11.540)	2.393 (− 0.839 to 5.624)	1.582 (− 1.072 to 4.236)
Without toilet (ref. no.)	− 0.362 (− 8.759 to 8.035)	− 1.134 (− 9.312 to 7.043)	0.181 (− 8.003 to 8.364)	2.187 (− 5.674 to 10.048)	** − 2.889 (− 5.170 to − 0.608)**	** − 1.891 (− 3.760 to − 0.021)**
Without shower (ref. no.)	− 0.362 (− 8.759 to 8.035)	− 1.134 (− 9.312 to 7.043)	0.181 (− 8.003 to 8.364)	2.187 (− 5.674 to 10.048)	** − 2.889 (− 5.170 to − 0.608)**	** − 1.891 (− 3.760 to − 0.021)**
Without washing machine (ref. no.)	4.888 (− 0.721 to 10.497)	2.248 (− 3.374 to 7.869)	− 4.831 (− 10.297 to 0.635)	− 1.309 (− 6.747 to 4.128)	** − 2.211 (− 3.736 to − 0.686)**	− 0.881 (− 2.202 to 0.440)
House dampness (ref. no.)	**4.396 (2.536 to 6.257)**	**3.741 (1.818 to 5.664)**	** − 5.103 (− 6.926 to − 3.279)**	** − 4.157 (− 5.999 to − 2.316)**	** − 0.666 (− 1.208 to − 0.124)**	− 0.092 (− 0.564 to 0.379)
Insufficient daylight (ref. no.)	**2.680 (0.002 to 5.358)**	**2.787 (0.095 to 5.479)**	** − 3.880 (− 6.478 to − 1.281)**	** − 3.124 (− 5.714 to − 0.534)**	− 0.325 (− 1.095 to 0.445)	0.297 (− 0.354 to 0.948)
Noise (ref. no.)	**1.584 (0.068 to 3.101)**	**1.793 (0.280 to 3.305)**	** − 2.159 (− 3.633 to − 0.685)**	** − 2.143 (− 3.600 to − 0.686)**	− 0.306 (− 0.735 to 0.123)	− 0.090 (− 0.452 to 0.271)
Pollution (ref. no.)	**2.947 (1.105 to 4.789)**	**2.580 (0.746 to 4.414)**	** − 2.274 (− 4.061 to − 0.486)**	** − 2.093 (− 3.860 to − 0.325)**	− 0.015 (− 0.543 to 0.513)	0.096 (− 0.349 to 0.541)
Violence (ref. no.)	**4.390 (2.193 to 6.588)**	**3.940 (1.723 to 6.157)**	** − 3.061 (− 5.211 to − 0.910)**	** − 2.819 (− 4.948 to − 0.691)**	− 0.428 (− 1.064 to 0.208)	− 0.001 (− 0.548 to 0.547)
Overcrowding (ref. no.)	**4.921 (1.131 to 8.710)**	3.751 (− 0.148 to 7.650)	− 2.063 (− 5.774 to 1.648)	− 1.405 (− 5.166 to 2.355)	− 0.173 (− 1.216 to 0.869)	0.291 (− 0.607 to 1.190)
Housing typology (ref. conventional)						
Social	0.374 (− 2.260 to 3.009)	0.826 (− 2.065 to 3.717)	− 1.854 (− 4.383 to 6.76)	− 0.642 (− 3.374 to 2.091)	** − 1.137 (− 1.848 to − 0.426)**	0.050 (− 0.619 to 0.719)
Affordable	2.306 (− 1.339 to 5.951)	1.300 (− 2.411 to 5.011)	** − 3.905 (− 7.366 to − 0.444)**	− 1.884 (− 5.356 to 1.588)	** − 1.100 (− 2.895 to − 0.139)**	− 0.107 (− 0.939 to 0.725)
Substandard housing (“Ilhas”)	− 0.124 (− 4.822 to 4.574)	0.973 (− 3.945 to 5.891)	− 1.988 (− 6.725 to 2.749)	− 2.998 (− 7.933 to 1.936)	** − 1.523 (− 2.895 to − 0.151)**	0.603 (− 0.631 to 1.836)
Housing costs: percentage of the income spent on housing (ref. less than 40%)	**2.341 (0.811 to 3.871)**	**2.001 (0.426 to 3.577)**	** − 3.185 (− 4.642 to − 1.729)**	** − 2.435 (− 3.930 to − 0.940)**	** − 0.731 (− 1.160 to − 0.302)**	− 0.194 (− 0.570 to 0.182)
Inability to pay rent/loan (ref. no.)	0.141 (− 11.712 to 11.995)	1.436 (− 10.108 to 12.980)	− 0.866 (− 12.417 to 10.686)	− 4.400 (− 15.495 to 6.696)	1.389 (− 1.847 to 4.625)	− 0.149 (− 2.801 to 2.503)
The landlord has increased the rent of the house to a very high value (ref. no.)	4.963 (− 0.982 to 10.908)	3.740 (− 2.090 to 9.570)	− 4.153 (− 9.950 to 1.645)	− 1.974 (− 7.598 to 3.649)	** − 2.658 (− 4.271 to − 1.046)**	** − 2.179 (− 3.516 to − 0.842)**
I got evicted (ref. no.)	**16.702 (4.932 to 28.472)**	**12.651 (0.852 to 24.450)**	− 9.230 (− 20.755 to 2.296)	− 3.406 (− 14.866 to 8.053)	** − 4.635 (− 7.848 to − 1.421)**	− 1.993 (− 4.782 to 0.796)
The lease was not renewed (ref. no.)	0.394 (− 8.003 to 8.791)	− 1.687 (− 9.937 to 6.562)	3.323 (− 6.114 to 12.759)	2.827 (− 6.230 to 11.883)	0.721 (− 1.925 to 3.367)	− 0.117 (− 2.280 to 2.046)
Residential moves (ref. not moved more than once)	**4.519 (1.888 to 7.150)**	**4.129 (1.542 to 6.716)**	− 2.114 (− 4.699 to 0.470)	− 2.217 (− 4.728 to 0.294)	− 0.439 (− 1.208 to 0.331)	− 0.612 (− 1.247 to 0.022)
Occupation regime (ref. owner)						
Renter	1.255 (− 0.454 to 2.965)	1.168 (− 0.596 to 2.931)	** − 2.176 (− 3.837 to − 0.516)**	− 1.389 (− 3.091 to 0.313)	** − 0.896 (− 1.363 to − 0.430)**	− 0.236 (− 0.650 to 0.178)
Lives in family housing	1.928 (− 2.963 to 6.819)	0.962 (− 4.252 to 6.175)	− 3.957 (− 8.680 to 0.766)	− 3.297 (− 8.292 to 1.698)	0.168 (− 1.106 to 1.441)	0.222 (− 0.927 to 1.371)
Other	5.311 (− 2.202 to 12.824)	7.843 (− 0.333 to 16.019)	− 4.359 (− 11.614 to 2.896)	− 3.987 (− 11.815 to 3.841)	− 0.082 (− 2.351 to 2.186)	0.853 (− 1.315 to 3.022)

Adjusted Coefficients (*β*) was adjusted for sex, age, marital status, education, and current or last professional activity; bold denotes statistically significant associations

*Ref* reference category

For QoL, individuals living in houses without heating (*β* =  − 1.942, 95% CI =  − 3.438 to − 0.445), with leaks/dampness/rot (*β* =  − 4.157, 95% CI =  − 5.999 to − 2.316), insufficient daylight (*β* =  − 3.124, 95% CI =  − 5.714 to − 0.534), neighborhood noise (*β* =  − 2.143, 95% CI =  − 3.600 to − 0.686), pollution/grime (*β* =  − 2.093, 95% CI =  − 3.860 to − 0.325) and violence/crime/vandalism (*β* =  − 2.819, 95% CI =  − 4.948 to − 0.691), and who experienced housing cost overburden (*β* =  − 2.435, 95% CI =  − 3.930 to − 0.940) reported lower QoL. While in the crude models, lower levels of QoL were found among those living in affordable housing [*β* =  − 3.905 (− 7.366 to − 0.444), reference: conventional housing] and renters [*β* =  − 2.176 (− 3.837 to − 0.516), reference: homeowner], and these associations lost significance after adjustment.

Older adults with no toilet [*β* =  − 1.891 (− 3.760 to − 0.021)] or shower [*β* =  − 1.891 (− 3.760 to − 0.021)] at home or who faced forced displacement due to rent increases [*β* =  − 2.179 (− 3.516 to − 0.842)] presented lower cognitive levels. Initially, crude models indicated that those without a private garden [*β* =  − 0.549 (− 1.062 to − 0.036)], heating [*β* =  − 1.081 (− 1.474 to − 0.688)], a balcony [*β* =  − 0.595 (− 1.138 to − 0.052)], or a washing machine [*β* =  − 2.211 (− 3.736 to − 0.686)]; those living in houses with leaks/dampness/rot [*β* =  − 0.666 (− 1.208 to − 0.124)], or in social [*β* =  − 1.137 (− 1.848 to − 0.426)], affordable [*β* =  − 1.100 (− 2.895 to − 0.139)], or substandard housing [*β* =  − 1.523 (− 2.895 to − 0.151)]; those facing housing cost overburden [*β* =  − 0.731 (− 1.160 to − 0.302)]; those evicted [*β* =  − 4.635 (− 7.848 to − 1.421)]; and renters [*β* =  − 0.896 (− 1.363 to − 0.430), reference: homeowner] were found to have lower levels of cognition. However, after adjustment, these associations lost statistical significance.

As depicted in Table 4, and even after adjustment, older adults in neighborhoods with pollution/grime [OR = 0.494 (0.322 to 0.756)] and with violence/crime/vandalism [OR = 0.477 (0.284 to 0.801)], those living in social housing [OR = 0.728 (0.575 to 0.922), reference: conventional housing], and those who moved more frequently [OR = 0.475 (0.257 to 0.879)] were less likely to have a good perception of healthy ageing.

**Table 4:** Associations between housing insecurity variables and perception of healthy ageing, sleeping hours, and sleep quality: EPIPorto Cohort, Porto, Portugal, (2022)

Exposures	Health outcomes					
	Perception of healthy ageing		Insufficient sleep duration (less than 7 h)		Poor sleep quality	
	Crude odds ratio (95% CI)	Adjusted odds ratio (95% CI)	Crude odds ratio (95% CI)	Adjusted odds ratio (95% CI)	Crude odds ratio (95% CI)	Adjusted odds ratio (95% CI)
Without private garden (ref. no.)	1.017 (0.781 to 1.762)	1.111 (0.727 to 1.698)	0.865 (0.570 to 1.315)	0.836 (0.540 to 1.293)	0.831 (0.528 to 1.310)	0.841 (0.519 to 1.363)
Without heating (ref. no.)	1.124 (0.814 to 1.553)	1.167 (0.811 to 1.680)	1.012 (0.726 to 1.409)	1.041 (0.711 to 1.522)	0.984 (0.693 to 1.398)	0.976 (0.648 to 1.468)
Without balcony (ref. no.)	1.061(0.676 to 1.665)	0.961 (0.596 to 1.548)	1.199 (0.771 to 1.866)	1.248 (0.782 to 1.993)	1.323 (0.811 to 2.158)	1.500 (0.882 to 2.554)
Without piped water (ref. no.)	1.048 (0.078 to 14.140)	1.335 (0.097 to 18.370)	1.350 (0.084 to 21.687)	1.516 (0.092 to 24.956)	0.475 (0.030 to 7.644)	0.526 (0.031 to 8.814)
Without toilet (ref. no.)	1.049 (0.166 to 6.624)	1.035 (0.160 to 6.680)	0.673 (0.061 to 7.463)	0.616 (0.054 to 6.984)	0.953(0.086 to 10.582)	1.015 (0.089 to 11.506)
Without shower (ref. no.)	1.049 (0.166 to 6.624)	1.035 (0.160 to 6.680)	0.673 (0.061 to 7.463)	0.616 (0.054 to 6.984)	0.953 (0.086 to 10.582)	1.015 (0.089 to 11.506)
Without washing machine (ref. no.)	0.573 (0.156 to 2.096)	0.446 (0.114 to 1.745)	0.807 (0.191 to 3.408)	0.876 (0.202 to 3.808)	0.792 (0.187 to 3.350)	0.602 (0.133 to 2.731)
House dampness (ref. no.)	0.675(0.440 to 1.037)	0.599(0.379 to 0.945)	0.776(0.496 to 1.214)	0.839 (0.521 to 1.351)	1.190 (0.740 to 1.912)	1.265 (0.753 to 2.124)
Insufficient natural light (ref. no.)	0.905 (0.493 to 1.661)	0.783 (0.416 to 1.473)	1.372 (0.750 to 2.509)	1.414 (0.750 to 2.662)	1.120 (0.582 to 2.154)	0.985 (0.494 to 1.964)
Noise (ref. no.)	0.721 (0.512 to 1.015)	0.708 (0.496 to 1.012)	1.334 (0.936 to 1.901)	1.319 (0.912 to 1.908)	0.945 (0.651 to 1.374)	0.917 (0.618 to 1.360)
Pollution (ref. no.)	**0.514 (0.341 to 0.777)**	**0.494 (0.322 to 0.756)**	**1.571 (1.018 to 2.424)**	1.491 (0.950 to 2.340)	1.039 (0.654 to 1.650)	1.111 (0.682 to 1.809)
Violence (ref. no.)	**0.497 (0.303 to 0.815)**	**0.477 (0.284 to 0.801)**	1.299 (0.776 to 2.173)	1.224 (0.710 to 2.110)	1.468 (0.820 to 2.627)	1.344 (0.732 to 2.469)
Overcrowding (ref. no.)	1.059 (0.440 to 2.548)	0.941 (0.377 to 2.352)	1.506 (0.629 to 3.605)	1.809 (0.696 to 4.699)	1.200 (0.458 to 3.144)	1.237 (0.426 to 3.593)
Housing typology (ref. conventional)						
Social	0.720 (0.397 to 1.303)	**0.728 (0.575 to 0.922)**	1.650 (0.938 to 2.902)	**2.155 (1.102 to 4.213)**	0.645 (0.362 to 1.147)	**0.445 (0.220 to 0.900)**
Affordable	0.834 (0.376 to 1.850)	0.971 (0.719 to 1.311)	1.043 (0.469 to 2.318)	1.307 (0.567 to 3.015)	**0.443 (0.201 to 0.979)**	**0.381 (0.162 to 0.896)**
Substandard housing (“Ilhas”)	2.471 (0.659 to 9.268)	1.123 (0.728 to 1.733)	0.889 (0.287 to 2.757)	1.091 (0.327 to 3.641)	2.437 (0.534 to 11.132)	2.033 (0.411 to 10.050)
Housing costs: percentage of the income spent on housing (ref. less than 40%)	0.264 (0.568 to 1.168)	0.752 (0.511 to 1.107)	1.161 (0.798 to 1.690)	1.253 (0.839 to 1. 871)	0.926 (0.625 to 1.372)	0.936 (0.611 to 1.433)
Inability to pay rent/loan (ref. no.)	0.282 (0.028 to 2.813)	0.297 (0.029 to 3.042)	1.340 (0.083 to 21.538)	1.067 (0.065 to 17.440)	< 0.001 (0.000 to + ∞)	< 0.001 (0.000 to + ∞)
The landlord has increased the rent of the house to a very high value (ref. no.)	0.480 (0.110 to 2.099)	0.494 (0.116 to 2.108)	1.345 (0.333 to 5.432)	1.517 (0.370 to 6.227)	0.815 (0.193 to 3.449)	0.754 (0.174 to 3.267)
I got evicted (ref. no.)	1,336,695,913.3 (0.000 to + ∞)	938,062,238.64 (0.000 to + ∞)	< 0.001 (0.000 to + ∞)	< 0.001 (0.000 to + ∞)	3,114,784,673.9 (0.000 to + ∞)	2,022,456,373.0 (0.000 to + ∞)
The lease was not renewed (ref. no.)	0.308 (0.034 to 2.815)	0.356 (0.037 to 3.429)	1.342 (0.188 to 9.594)	1.430 (0.195 to 10.486)	1.476 (0.152 to 14.286)	1.438 (0.143 to 14.502)
Residential moves (ref. not moved more than once)	**0.498 (0.272 to 0.910)**	**0.475 (0.257 to 0.879)**	0.914 (0.498 to 1.678)	0.953 (0.512 to 1.775)	0.912 (0.486 to 1.713)	0.849 (0.440 to 1.638)
Occupation regime (ref. owner)						
Renter	0.767 (0.520 to 1.132)	0.867 (0.747 to 1.007)	1.063 (0.719 to 1.571)	1.151 (0.750 to 1.769)	1.123 (0.740 to 1.705)	1.024 (0.641 to 1.635)
Lives in family housing	1.118 (0.390 to 3.204)	1.031 (0.675 to 1.575)	0.289 (0.082 to 1.019)	0.362 (0.099 to 1.318)	3.764 (0.849 to 16.691)	2.847 (0.617 to 13.143)
Other	1.002 (0.160 to 6.263)	0.816 (0.407 to 1.636)	5.386 (0.597 to 48.610)	8,943,336,327.3 (0.000 to + ∞)	0.335 (0.055 to 2.026)	0.170 (0.017 to 1.690)

Adjusted odds ratio was adjusted for sex, age, marital status, education, and current or last professional activity; bold denotes statistically significant associations

*Ref* reference category

Finally, concerning sleeping hours, older adults living in neighborhoods with pollution/grime [OR = 1.571 (1.018 to 2.424)] were more likely to sleep for less than 7 h (insufficient sleep); nonetheless, this association disappeared in the adjusted model. In turn, after adjusting for confounders, those living in social housing were more likely to report insufficient sleep [OR = 2.155 (1.102 to 4.213)]. Unexpectedly, poor sleep quality was least likely both among those living in social housing [OR = 0.445 (0.220 to 0.900), reference: conventional housing] and affordable housing [OR = 0.381 (0.168 to 0.896), reference: conventional housing] (Table 4).

## Discussion

Our study aimed to investigate the association between housing insecurity and indicators of older adults’ health and well-being living in Porto. We found that housing insecurity influences loneliness, QoL, cognitive function, and sleep in the sense that the lack of stable, safe, adequate, and affordable housing leads to worse health and well-being outcomes.

Our findings indicate that individuals lacking home heating, experiencing leaks/dampness/rot in the house, insufficient daylight, noise, pollution/grime, or violence/crime/vandalism in their neighborhood are more likely to report loneliness and a worse QoL. Indoor cold can affect social perceptions making people long for social connections [21]. Having dampness at home can cause physical discomfort and psychological distress influencing people’s moods, hindering social connections and increasing isolation. Additionally, light is known to play a crucial factor in mood regulation since its presence influences the levels of neurotransmitters like serotonin, the neurotransmitter of overall well-being and happiness [22]. Exposure to traffic noise has been linked to mental health issues and psychological disorders, including depression and anxiety [23]. Living in areas with higher pollution levels is also associated with a greater negative impact on mental well-being [24]. Again, feeling mentally unwell might hinder social interactions and, therefore, feelings of loneliness. Individuals exposed to violence might be restrained from feeling secure, which can make them have fewer interactions with close network members, perceive less social support from friends, and experience higher levels of loneliness [25]. Moreover, inadequate housing conditions can lead to adverse health effects, consequently compromising QoL [26].

Furthermore, we found that loneliness strongly increases when individuals are forced to move due to eviction compared to those not evicted. This aligns with previous research [27] and findings from a qualitative study conducted within the same city and cohort [11]. Eviction can lead to the loss of social ties and community support, making individuals feel more isolated as forming new bonds can be a long process. Our cross-sectional findings should be supplemented with longitudinal studies to explore the potential severe understudied health impacts of eviction.

Experiencing loneliness and a worse QoL was more common among those spending 40% or more of their income on housing (housing cost overburden). High housing costs strain other life aspects, making it difficult to afford essentials like healthcare, food, and education [16]. This can diminish QoL, elevate stress, anxiety, and depression and lead to negative emotions like fear, anger, resentment, and loneliness [28]. In our study, 28% of older adults face housing cost overburden, much higher than the 5% reported by Statistics Portugal from 2023 EU-SILC data (5%). This difference may be due to high housing costs in gentrifying cities like Porto and older adults’ limited disposable income compared to the overall adult population.

Older adults who faced forced displacement due to rent increases scored lower on the MMSE. While housing affordability’s impact on children’s cognitive function is well-studied, its effects on older adults remain understudied, a gap our study addresses. It is plausible that rising rent prices might cause financial stress and anxiety, factors associated with cognitive decline [18].

Our study indicates that living in neighborhoods with pollution/grime and violence/crime/vandalism, as well as living in social housing, and moving house are associated with lower odds of a more positive perception of healthy ageing. Growing research shows that the environment around us influences ageing outcomes, contributing to diverse health outcomes among older adults of the same age. For instance, air and noise pollution have adverse effects on subjective well-being and health [29], perceived violence in the neighborhood is associated with poorer self-rated health [30], and residential stability was linked to better self-rated health [31].

Shorter sleep duration and poorer sleep quality were more common among individuals living in social housing. Similarly, a worse sleep quality is observed in residents in affordable housing. This might be explained by neighborhood disorder and perceived building problems experienced in social housing that may contribute to sleep disturbances and poor sleep quality [32].

The present study has some limitations. Given the cross-sectional design, causation cannot be inferred from these findings. We evaluated our variables at a single point so that we cannot rule out the possibility of reverse causation. Therefore, while the relationships observed are plausible from a biological and public health perspective, they should be interpreted cautiously. Longitudinal studies are needed to determine the directionality of these associations. Moreover, the small number of observations in some evicted-related categories reduced our statistical power, as mirrored in the large confidence intervals.

Despite these limitations, our study has important strengths. We assessed various dimensions of housing insecurity and their potential health implications in the general older adult population. We believe these findings are particularly notable among an older population, as loneliness, quality of life, cognitive function, perceptions of ageing, and sleep are interconnected and significantly impact the overall well-being of older adults. Addressing these issues is crucial given that older adults face an increased risk for loneliness—currently considered an under-addressed and growing epidemic [33]—experience intrinsic age-related cognitive [34] and physical declines and are vulnerable to the effects of sleep disturbances on well-being. Housing insecurity is especially concerning because it may exacerbate these age-related health declines, further compromising the well-being and quality of life of older individuals as they age. Health outcomes were evaluated by trained technicians using validated scales, enhancing the validity and comparability of our findings. Finally, this research is pioneering in Porto, as no prior study has explored how the different aspects of housing insecurity affect the health of older adults.

As discussed, housing insecurity can negatively impact the health and well-being of older adults. Therefore, there should be a public health investment in giving older adults access to stable, safe, adequate, and affordable housing. Housing programs that provide the necessary conditions to make house modifications to comply with the needs of older adults, policies to create affordable housing, and policies to protect against abusive tactics of landlords should be nudged to mitigate health disparities in this population [35]. In this specific population, opportunities should be given so that older people can age in place, that is, to remain in their own homes or neighborhoods with which they are familiar to make the process easier and cause less disruption in their lives.

## Conclusion

Housing insecurity appears to bring potential threats to older adults’ health and well-being. The most prominent and consistent consequences were higher levels of loneliness and a worse QoL. Cognitive function, perceived healthy ageing, and sleep were related to fewer components of housing insecurity. The findings support the need for good quality, affordable, and stable housing to promote healthy ageing in a person’s desired residential location.

## Supplementary Information

Below is the link to the electronic supplementary material.

Supplementary file1 (DOCX 78 KB)

## Data Availability

The data underpinning the study findings is restricted since they were used under authorization for this specific study and are not publicly accessible.
