# Supplementary material for: Housing Insecurity and Older Adults’ Health and Well-Being in a Gentrifying City: Results from the EPIPorto Cohort Study
Source: J Urban Health. 2024 Sep 26;102(1):19–34. doi: 10.1007/s11524-024-00921-4 (PMC11865402; doi:10.1007/s11524-024-00921-4)
Supplement: Supplementary file 1 — Supplementary file1 (DOCX 78 KB) [file 11524_2024_921_MOESM1_ESM.docx]

**
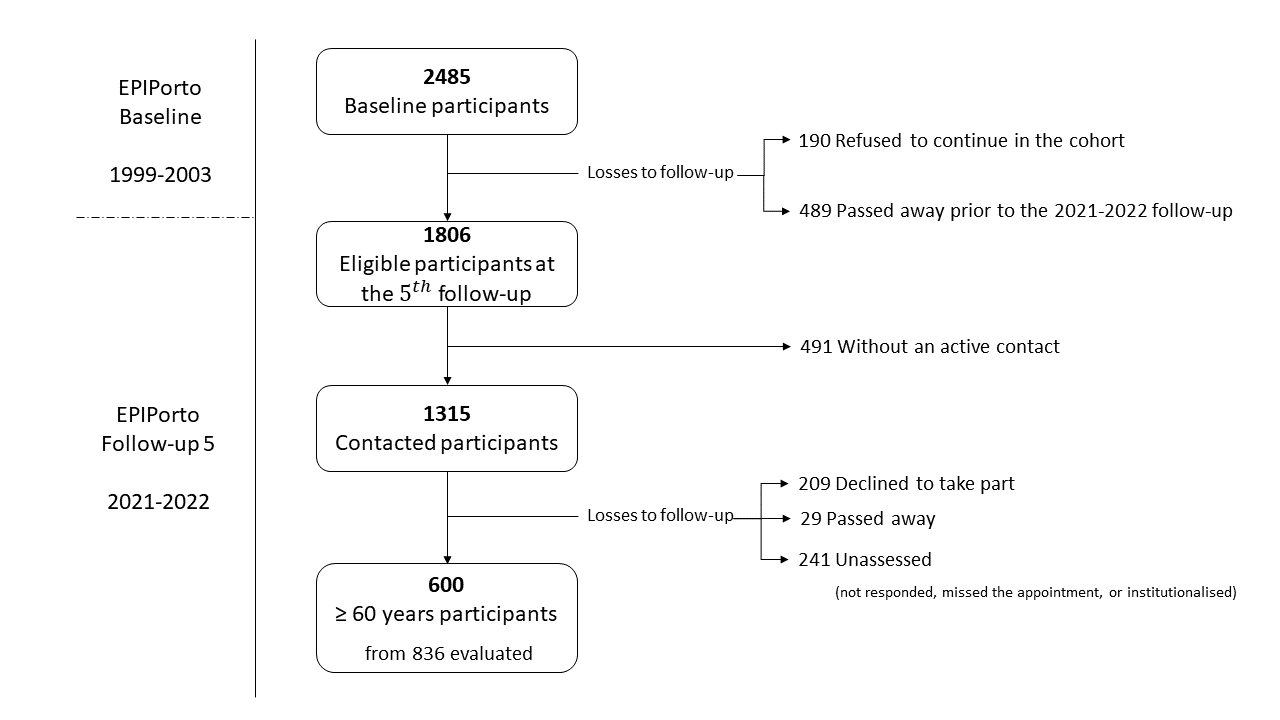
Supplementary material**

Figure S1: Flow-chart describing the selection of the studied sample.


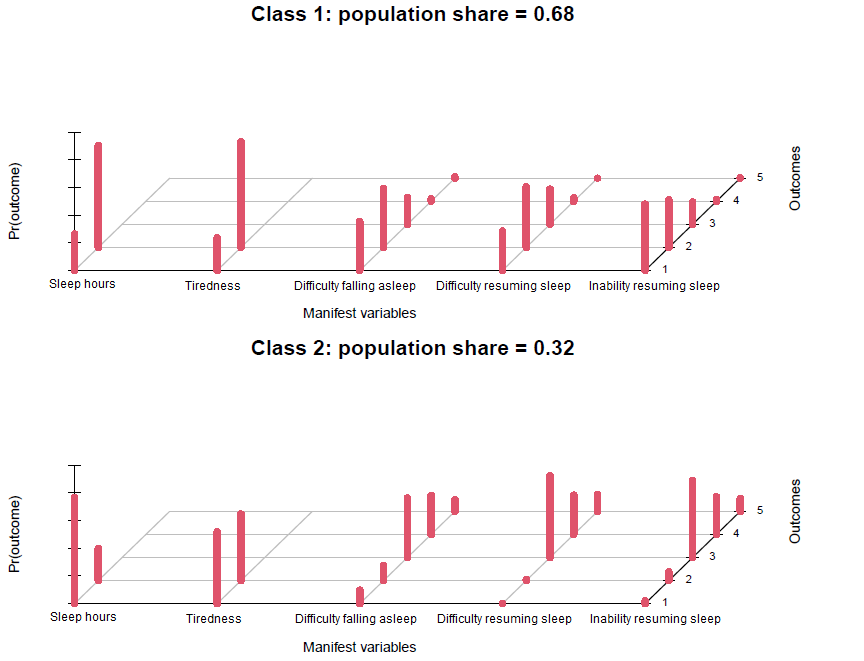


Figure S2: Graph showing the two latent classes of sleep quality among participants: 'bad sleep quality' (Class 1) and 'good sleep quality' (Class 2). These classes are based on responses to the following questions about sleep habits: 'How many hours do you sleep on average per night?', 'Do you often feel tired, fatigued, or sleepy during the day?', 'How often in the last month have you had difficulty falling asleep?', 'How often in the last month have you woken up and had difficulty falling back asleep?', and 'How often in the last month did you wake up before your desired time and couldn't fall back asleep?'"

Tabela S1: Comparison of age [one-way analysis of variance (ANOVA)], sex, and level of education [chi-square test for independence] of participants from the fifth evaluation of EPIPorto study (n=600) with those who abstained from participation: refuses (n=178) and non-participants (n=1240).

|  | **Participations (n=600)** | **Refuses (n=178)** | **Non-participants (n=1240)** | **p-value** |
| --- | --- | --- | --- | --- |
| Mean age (range) | 73.14 (60-99) | 78.93 (61-95) | 84.55 (60-116) | <0.001 |
| Sex |  |  |  |  |
| Female | 390 (65.0%) | 122 (68.5%) | 736 (59.4%) | 0.010 |
| Male | 210 (35.0%) | 56 (31.5%) | 504 (40.6%) |  |
| Level of education* |  |  |  |  |
| Primary | 251 (41.8%) | 111 (62.4%) | 827 (66.7%) | <0.001 |
| Secondary | 159 (26.5%) | 40 (22.5%) | 237 (21.6%) |  |
| Tertiary | 190 (31.7%) | 27 (15.2%) | 1. (14.2%) |  |

* Primary education was defined as completing at least eight years of schooling, secondary education was defined as completing nine to 12 years, and tertiary education was described as completing more than 12 years.
